# Paramedic powers in mental health crises: A comparative legal analysis

**DOI:** 10.1177/00048674251395412

**Published:** 2025-12-07

**Authors:** Dylan A Mordaunt, David O’Byrne, Nicole Jones

**Affiliations:** 1 Faculty of Education, Health and Psychological Sciences, Victoria University of Wellington, Wellington, New Zealand; 2 Wellington Free Ambulance, Wellington, New Zealand; 3 Hato Hone St John, Auckland, New Zealand

**Keywords:** Mental health law, paramedics, suicide prevention, pre-hospital care, emergency medical services, health policy, interagency collaboration, comparative policy analysis, police withdrawal

## Abstract

**Introduction:**

Effective management of mental health crises is a growing global concern, significantly impacting emergency services. In New Zealand the New Zealand Police have begun reducing their involvement in mental health emergencies. This shift positions paramedics as primary responders in pre-hospital mental health crisis management. This current study conducts a comparative analysis of mental health legislation in New Zealand, Australian jurisdictions and the United Kingdom to assess how laws empower paramedics in mental health crises.

**Methods:**

A structured framework was employed to evaluate 12 key domains relevant to pre-hospital mental health interventions. These domains include criteria for involuntary detention, emergency detention and transportation powers, integration of services and legal protections for paramedics.

**Results:**

The analysis reveals that New Zealand’s Mental Health Bill (as introduced in 2024) emphasises reducing coercion and promoting culturally appropriate care but lacks provisions granting paramedics the authority to manage crises in isolation. In contrast, jurisdictions like the Northern Territory, Western Australia and Queensland empower paramedics with greater legal authority and more integrated roles in mental health emergencies.

**Discussion:**

The absence of health-based legal tools and insufficient integration with mental health services in New Zealand may limit paramedics’ effectiveness in crisis management, potentially increasing reliance on police and delaying interventions. Recommendations include expanding paramedic authority in line with other jurisdictions and improving integration with mental health services. By adopting models from leading Australian jurisdictions, New Zealand paramedics will be better placed to manage mental health responses and support a reduction in police involvement.

## Introduction

Mental health crises, including suicide attempts, are a growing concern globally, placing increasing demands on emergency services (World Health Organization, 2021). For those with lived experience of mental distress, the nature of the first response can have a profound and lasting impact on their willingness to engage with services in the future. The evolution of paramedicine from a transport-focused service to a clinical profession with a wide scope of practice has positioned paramedics as key decision-makers in the pre-hospital setting. However, legislative frameworks governing responses to these crises vary across jurisdictions, influencing the effectiveness of pre-hospital care and creating significant downstream consequences for the entire health system (Emond et al., 2019; Rees et al., 2017; Sipe et al., 2015; Townsend and Luck, 2009). In New Zealand, a significant policy shift is imminent: from 4 November 2024, the New Zealand Police reduced their involvement in mental health emergencies, focusing solely on situations involving criminal offences or serious safety risks (New Zealand Police, 2024). This policy change, while well intentioned, presents both challenges and opportunities for mental health and ambulance services. It firmly places paramedics as the default primary responders in these complex situations.

The New Zealand Mental Health Bill (as introduced in 2024, hereafter the ‘2024 Bill’) seeks to modernise mental health care by promoting a rights-based approach and reducing coercion (New Zealand Ministry of Health, 2020; New Zealand Parliament, 2024). This follows an earlier draft and a public submission process (New Zealand Ministry of Health, 2021). However, analysis of the Bill reveals a significant policy gap: while it rightly seeks to decriminalise the mental health response, it does not concurrently empower the health-based workforce intended to fill the void. Questions therefore arise about whether paramedics are sufficiently empowered and supported to manage this new responsibility, notwithstanding the need to resource New Zealand ambulance services adequately (Augustus and Patrick, 2022; Marks, 2022). While the Bill grants paramedics specific powers, such as the authority to sedate individuals under certain conditions (Clause 184), it does not broadly address their legal authority in other aspects of mental health crises, nor does it provide for expanded clinical roles beyond this specific power (Best and Taylor, 2021; New Zealand Ministry of Health, 1981). This limited explicit legislative empowerment raises a question of whether this omission is by design or an oversight, and how other comparable jurisdictions have navigated this complex issue. The answer has implications for psychiatric services, which bear the brunt of a poorly coordinated pre-hospital system through avoidable presentations to emergency departments (EDs).

This article provides a comprehensive comparative analysis of the legislative frameworks of New Zealand’s 2024 Bill with those of all Australian jurisdictions – specifically the Australian Capital Territory (2015), New South Wales (2007), Northern Territory (1998), Queensland (2016), South Australia (2009), Tasmania (2013), Victoria (2022) and Western Australia (2014) – and the United Kingdom’s Mental Health Act (2022). The evaluation focuses on assessing the potential of these laws to reduce coercion, decrease police involvement and empower frontline clinicians to manage out-of-hospital mental health crises safely and effectively, ultimately aiming to inform evidence-based policy reform in New Zealand (Davidson et al., 2016; Perlin, 2019).

## Methods

### Design

**Figure 1.**
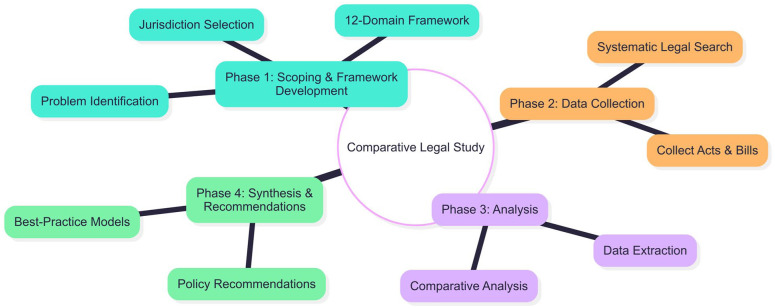
Scientific workflow of the comparative analysis.

A comparative policy analysis was conducted to assess mental health laws and bills from multiple jurisdictions, including New Zealand, the United Kingdom and all Australian states and territories. The overall scientific workflow for this study is depicted in Figure 1.

The analysis focused on key domains relevant to pre-hospital mental health crisis interventions from an ambulance service and paramedic perspective. These jurisdictions were selected due to their shared common law heritage and similar parliamentary and healthcare systems, which provides a relevant and coherent context for comparison. While other jurisdictions, such as the United States or Canada, have valuable perspectives, their significantly different legal and healthcare funding models would necessitate a separate and more extensive analysis beyond the scope of this study.

### Framework

A comparative framework was developed to evaluate each jurisdiction’s mental health laws, focusing on 12 specific domains. These were formulated based on key questions arising from the operational and legal challenges anticipated from the police withdrawal from New Zealand mental health services. The guiding questions for each domain are detailed in Appendix 1.

### Data sources

The mental health laws reviewed were obtained from publicly available legal databases. The primary documents were the New Zealand Mental Health Bill (as introduced to Parliament in 2024) and the preceding Mental Health (Compulsory Assessment and Treatment) Act 1992 (New Zealand Ministry of Health, 2024; New Zealand Parliament, 1992). Australian state and territory legislation was also reviewed (Australian Capital Territory Legislative Assembly, 2015; New South Wales Parliament, 2007; Northern Territory Parliament, 1998; Queensland Parliament, 2016; South Australia Parliament, 2009; Tasmania Parliament, 2013; Victoria Parliament, 2022; Western Australia Parliament, 2014). In addition, the United Kingdom’s Mental Health Act (2022) was analysed (United Kingdom Parliament, 2022).

### Analysis

Each jurisdiction’s legal provisions were analysed according to the comparative framework. The analysis involved a detailed examination of how each law addresses the 12 key domains. The findings were systematically compared to highlight differences and similarities in paramedic roles, legal protections and integration with mental health services. This comparative approach allowed for the identification of key areas of interest and potential best practices that could inform the expansion of paramedic roles in New Zealand’s mental health crisis response system.

## Results

**Figure 2.**
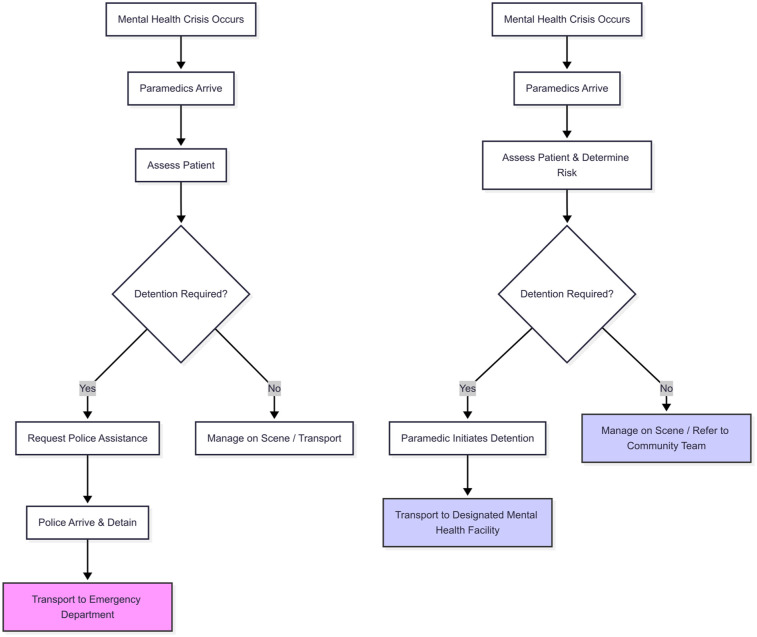
Comparative workflow for paramedic response in mental health crises.

The comparative analysis reveals significant variations in the legislative frameworks governing pre-hospital mental health crisis response across the selected jurisdictions. The findings are detailed in the Supplementary Document and summarised below across three key themes: the legal authority and role of paramedics; the integration of ambulance and mental health services; and the approaches to patient rights, coercion and cultural safety. The difference in paramedic workflow between New Zealand and a jurisdiction with empowered paramedics, such as the Northern Territory, is illustrated in Figure 2.

### Paramedic authority and role

A key differentiator between jurisdictions is the extent of legal authority granted to paramedics. In New Zealand, under the 1992 Act, paramedics have a limited role, primarily focused on transport without any independent authority to detain individuals for assessment. The proposed 2024 Bill introduces a specific power for paramedics to sedate individuals under certain conditions (Clause 184), which is a significant independent authority. However, this does not extend to a general power to detain for assessment. This creates a situation where a paramedic in Wellington, faced with a person who is clearly unwell and at risk but refusing transport, and where sedation is not appropriate or effective, may still need to call for police back-up, potentially escalating the situation and causing significant delays. This contrasts sharply with jurisdictions like the Northern Territory, where a paramedic in Darwin, faced with the same situation, is empowered by statute to make a clinical determination of risk and, if necessary, independently and lawfully detain the person for transport to a mental health facility.

Other jurisdictions offer a spectrum of intermediate powers. In Western Australia and the ACT, paramedics can issue specific transport or apprehension orders in emergencies, providing a clear legal basis for action without requiring police presence. Queensland enables a sophisticated collaborative model where designated, experienced paramedics can be certified as ‘authorised mental health practitioners’, allowing them to guide and authorise actions for police and other responders. In contrast, the models in Victoria, New South Wales and the UK position paramedics within integrated teams or under the direction of mental health clinicians or police. Here, they act in a supportive capacity, contributing their medical skills but not as the primary autonomous decision-makers regarding detention.

Consequently, the legal protections afforded to paramedics vary significantly. The Northern Territory and Western Australia provide strong, explicit legal safeguards and immunities for paramedics exercising their legislated mental health powers in good faith. In contrast, New Zealand offers limited specific legal protection, leaving paramedics to rely on the ambiguous and high-stakes interpretation of general provisions of criminal law (such as section 41 of the Crimes Act 1961) when intervening to prevent suicide Mordaunt et al., (2025).

### System integration and collaboration

The integration of ambulance services with the broader mental health system is another area of divergence. Victoria and Western Australia demonstrate strong integration, with paramedics forming part of multidisciplinary crisis response teams (such as Victoria’s PACER model) that collaborate closely with mental health clinicians, often in the same vehicle. This allows for on-the-spot specialist assessment and referral. The UK model also promotes effective coordination through the National Health Service (NHS), where ambulance trusts are a core component of the public health system.

In New Zealand, however, the integration is less developed. The 2024 Bill promotes integrated care conceptually, but the structural disconnection between the privately operated ambulance services and the public mental health system presents substantial practical barriers. This is compounded by a lack of public governance and funding mechanisms for ambulance services to support the extended on-scene times and complex care navigation required for mental health crises Mordaunt (2025). This contrasts with the publicly funded and integrated systems in Victoria and the Northern Territory, which are structured to facilitate more cohesive and flexible emergency responses.

### Rights, coercion and cultural safety

There is a clear and commendable trend across most jurisdictions towards minimising coercion and promoting patient rights. The New Zealand 2024 Bill, along with legislation in the United Kingdom, Victoria and Western Australia, places a strong emphasis on voluntary care and using involuntary measures only as a last resort. This marks a significant philosophical shift from the older, more paternalistic legislation like New Zealand’s 1992 Act.

The New Zealand 2024 Bill also has a strong focus on cultural appropriateness, particularly for Māori, mandating the consideration of cultural values and the involvement of whānau (family). This is mirrored in Western Australia and Victoria with respect to Aboriginal and Torres Strait Islander populations, where specific cultural liaison roles are often embedded in crisis services. However, a key weakness in the New Zealand legislation is that the operationalisation of these principles within the ambulance sector is not clearly defined. There is little guidance on how a paramedic, often working alone or with one other colleague, can meaningfully enact these principles without specific training and system support. Furthermore, oversight mechanisms for paramedics in mental health interventions are not well-defined in New Zealand, unlike in Victoria, Western Australia and the United Kingdom, where paramedics are accountable for their mental health-related decisions within clear clinical governance and integrated health service frameworks.

## Discussion

The impending withdrawal of police from most mental health responses in New Zealand creates an urgent need to ensure the legal and operational frameworks are fit for purpose. Our analysis shows that while the 2024 Bill makes positive strides in rights-based care, it leaves a critical gap by not empowering paramedics, who will become the de facto primary responders. This section discusses the key implications of this policy misalignment, focusing on paramedic capabilities, the risks of legal ambiguity, the challenges of system integration and the importance of a rights-based approach to care.

### Paramedic capabilities and policy misalignment

The modern paramedic is a registered health clinician with a wide scope of practice, operating across primary, urgent and emergency situations. Their education and training, typically to a bachelor’s degree level, equip them with skills in advanced assessment, diagnostics, complex clinical decision-making and the administration of a wide range of medications. However, under New Zealand’s current legislation (the 1992 Act) and the proposed 2024 Bill, while paramedics are granted a specific power to sedate individuals under certain conditions (Clause 184), their role remains largely confined to transport, lacking broader authority to detain individuals or make other independent clinical decisions in mental health crises. This is a profound underestimation of their capabilities and a misuse of a highly trained and capable workforce.

This policy misalignment is particularly acute for advanced or specialist paramedics who possess postgraduate qualifications and skills in de-escalation, advanced mental status examinations and navigating complex alternative care pathways. Relegating these senior clinicians to a simple transport role is not only inefficient but also fails to leverage their expertise to provide the best possible care in the community. Furthermore, this situation can lead to significant ‘moral distress’ – the psychological impact on clinicians who know the most appropriate clinical action but are prevented from taking it by systemic or legal barriers. They are left with the responsibility for the patient’s safety but without the requisite authority to act, creating a stressful and ethically challenging environment that can contribute to burnout and workforce attrition.

### The perils of legal ambiguity: relying on the crimes act

The most significant concern arising from this analysis is the lack of a comprehensive, health-focused legal framework for paramedic intervention, forcing a reliance on general criminal law for many critical situations Mordaunt (2025). While the 2024 Bill grants paramedics the specific power to sedate individuals under certain conditions (Clause 184), this does not extend to broader powers of detention or assessment. Consequently, for interventions beyond sedation, paramedics must still rely on section 41 of the Crimes Act 1961 to intervene to prevent suicide. This provision, available to any member of the public, remains an inadequate and potentially dangerous substitute for a comprehensive statutory power tailored to mental health crises.

First, the legal threshold for using this power is high and ill-defined for clinical practice. It requires a belief that the person is at an *immediate* risk of suicide, a term that is not clearly defined and may not cover a person who is in acute crisis and poses a significant risk to themselves, but whose actions are not imminent. This leaves paramedics in a legal grey area, having to make a high-stakes legal judgement rather than a clinical one.

Second, this ambiguity creates a significant ‘chilling effect’ on clinical practice. Faced with the possibility of acting unlawfully and potentially facing criminal charges for assault or civil liability, a paramedic may hesitate to intervene. This hesitation, born of legal uncertainty, can lead to delays in care and an escalation of the crisis, ultimately resulting in worse outcomes for the patient and potentially requiring the very police involvement the policy seeks to avoid.

Finally, using a criminal law provision to manage a health crisis is philosophically and therapeutically inappropriate. The purpose of the Crimes Act is to ensure public safety and assign culpability, whereas the purpose of mental health legislation is to provide care and treatment. Framing a health intervention through a criminal lens can damage the therapeutic alliance from the outset, making the person feel like they are being punished rather than cared for.

### System integration and downstream consequences

Effective crisis care depends on a highly integrated system, yet the New Zealand model creates significant barriers. The structural disconnection between the largely privately operated ambulance services and the public mental health system is a major obstacle. Unlike in Victoria, where the publicly funded PACER model pairs a paramedic with a mental health clinician in a dedicated response vehicle, the New Zealand system is fragmented. This makes seamless communication, shared decision-making and on-the-spot referrals to community-based alternatives extremely difficult.

The downstream consequences of this fragmentation are severe, particularly for EDs. When paramedics lack the legal authority and the integrated pathways to ‘see and treat’ or ‘see and refer’ a person in the community, the ED often becomes the only viable destination. This leads to several negative outcomes: (1) ED Overcrowding: It contributes to the overcrowding of EDs, increasing wait times for all patients; (2) Inappropriate Environment: The chaotic, brightly lit and noisy environment of an ED is often counter-therapeutic for a person in mental distress, potentially exacerbating their condition; and (3) Strain on Acute Mental Health Services: This influx of undifferentiated mental health presentations places an enormous burden on on-call hospital psychiatric liaison services, who must then assess patients that might have been more appropriately managed in the community.

Addressing this requires significant reform of the funding and contracting models for ambulance services. Contracts need to be revised to support longer on-scene times and to incentivize the use of alternative care pathways, such as crisis cafes or community hubs, rather than simply rewarding transport to an ED. This would require a shift from a fee-for-transport model to a model that funds the delivery of appropriate care, regardless of location. Furthermore, establishing clear clinical governance structures that span both ambulance and mental health services is essential for ensuring quality, safety and accountability.

### Rights, coercion and the therapeutic alliance

While the 2024 Bill’s focus on rights and minimising coercion is a major step forward, its effectiveness is still undermined by the lack of a comprehensive empowered health response. Although paramedics are granted the specific power to sedate individuals under certain conditions (Clause 184), this limited authority means that for other critical interventions, reliance on law enforcement may still be necessary. A key principle of modern, recovery-oriented mental health care is the development of a therapeutic alliance built on trust and collaboration. A response led primarily by a health professional (a paramedic) is far more conducive to building this alliance than a response that involves law enforcement. The continued, even if reduced, presence of police for certain mental health crises, no matter how well-intentioned, can be inherently intimidating and perceived as punitive, potentially damaging the therapeutic relationship before it has even begun.

Furthermore, the Bill’s emphasis on cultural competence, particularly for Māori, is difficult to operationalise without a properly equipped workforce. True cultural safety goes beyond simple awareness; it requires the time and skill to engage in whakawhanaungatanga (relationship building) and to understand concepts like taha wairua (spiritual health). A paramedic who is rushed, legally uncertain and lacking pathways to community-based Māori health providers will struggle to provide care that is genuinely culturally safe and respectful, regardless of the legislative intent. Empowering paramedics with the time, training and legal authority to de-escalate situations and make considered clinical decisions is, therefore, a prerequisite for fulfilling the rights-based ambitions of the 2024 Bill.

### Strengths and limitations

This study provides a timely and comprehensive comparative analysis of a critical and evolving policy area. By using a structured framework across multiple, comparable jurisdictions, it offers clear, evidence-based insights. However, the analysis is primarily based on legislative documents and does not capture the full complexity of on-the-ground implementation, which can vary. Future research could usefully explore the operational realities and outcomes of these different legislative models, for example, by examining coronial inquest findings related to pre-hospital mental health events.

### Recommendations

To address the identified gaps, we recommend a phased approach to reform in New Zealand. First, legislative reform is needed to provide paramedics with clear legal authority to detain, transport and assess individuals in mental health emergencies, similar to models in the Northern Territory or Queensland. This should include specific provisions for legal protection and indemnity for paramedics acting in good faith. Second, system integration must be improved through formal partnerships and revised funding models that support co-response teams and alternative care pathways, breaking down the silos between ambulance and mental health services. Third, workforce development is critical, requiring a national training framework for paramedics focusing on advanced assessment, de-escalation, risk management and culturally safe practices.

## Conclusion

In conclusion, the New Zealand Mental Health Bill (2024) is a positive and necessary step towards a more rights-based mental health system. However, in its current form, it leaves the primary health responders – paramedics – ill-equipped for their expanded role. By learning from the legislative and operational frameworks in leading Australian jurisdictions, New Zealand has a clear opportunity to build a more effective, safe and patient-centred crisis response system that truly supports those in their time of greatest need.

## Supplemental Material

sj-docx-2-anp-10.1177_00048674251395412 – Supplemental material for Paramedic powers in mental health crises: A comparative legal analysis Supplemental material, sj-docx-2-anp-10.1177_00048674251395412 for Paramedic powers in mental health crises: A comparative legal analysis by Dylan A Mordaunt, David O’Byrne and Nicole Jones in Australian & New Zealand Journal of Psychiatry

sj-pdf-1-anp-10.1177_00048674251395412 – Supplemental material for Paramedic powers in mental health crises: A comparative legal analysis Supplemental material, sj-pdf-1-anp-10.1177_00048674251395412 for Paramedic powers in mental health crises: A comparative legal analysis by Dylan A Mordaunt, David O’Byrne and Nicole Jones in Australian & New Zealand Journal of Psychiatry

sj-tex-3-anp-10.1177_00048674251395412 – Supplemental material for Paramedic powers in mental health crises: A comparative legal analysis Supplemental material, sj-tex-3-anp-10.1177_00048674251395412 for Paramedic powers in mental health crises: A comparative legal analysis by Dylan A Mordaunt, David O’Byrne and Nicole Jones in Australian & New Zealand Journal of Psychiatry
