# Supplementary material for: Paramedic powers in mental health crises: A comparative legal analysis
Source: Aust N Z J Psychiatry. 2025 Dec 7;60(2):184–90. doi: 10.1177/00048674251395412 (PMC12831802; doi:10.1177/00048674251395412)
Supplement: sj-docx-2-anp-10.1177_00048674251395412 – Supplemental material for Paramedic powers in mental health crises: A comparative legal analysis [file sj-docx-2-anp-10.1177_00048674251395412.docx]

# Supplementary Tables: Comparative Analysis of Mental Health Legislation

This document provides a detailed comparison of mental health legislation across New Zealand, various Australian jurisdictions, and the United Kingdom. The analysis is structured around 12 key domains relevant to the role of paramedics in pre-hospital mental health crisis response.

## Table 1: Detailed Comparative Analysis

| **Framework Element** | **New Zealand (2024 Bill - Updated Version)** | **Victoria (AUS)** | **NSW (AUS)** | **WA (AUS)** | **QLD (AUS)** | **TAS (AUS)** | **SA (AUS)** | **NT (AUS)** | **ACT (AUS)** | **UK (2022 Act - Updated Version)** | **Key Considerations** |
| --- | --- | --- | --- | --- | --- | --- | --- | --- | --- | --- | --- |
| **1. Criteria for Involuntary Detention** | Risk-based, rights-focused. Emphasises least restrictive environment. | Least restrictive, voluntary first. | Similar to NZ. | Risk-based, with specific powers for paramedics to detain. | Risk-based, requires examination by authorised practitioner. | Medical authority needed. | Similar to NSW. | Paramedics can detain based on reasonable belief of mental illness and risk. | Risk-based, focused on immediate risk of harm. | Risk-based. | Ensure paramedics have clear decision-making powers in NZ. |
| **2. Emergency Detention & Transportation** | Paramedics lack detention authority; can only transport. | Transport under direction of mental health teams. | Police assist with detention. | Paramedics can detain and transport to a designated mental health facility. | Authorised paramedics can make recommendations for transport; police can apprehend on that basis. | Paramedics assist clinicians. | Police assist. | Paramedics can detain and transport independently. | Authorised paramedics can apprehend and transport in emergencies (suicide/harm risk). | Police assist, paramedics transport. | Expand paramedic authority in NZ. |
| **3. Integration of Services** | Promotes integrated care, but lacks operational detail. | Strong integration in crisis teams (PACER model). | Police-clinician collaboration. | Multidisciplinary crisis teams (MCTs). | Strong integration via “Authorised Mental Health Services” model. | Requires closer collaboration. | Health service integration. | Full paramedic integration into mental health response. | Legally integrated via tribunal (ACAT) processes, but less operational integration of pre-hospital teams. | Strong health system integration. | Strengthen integration in NZ, resource ambulance services to employ mental health clinicians. |
| **4. Role of Paramedics vs Police** | Paramedics to take lead, but without specific powers. | Paramedics in integrated teams. | Police-heavy interventions. | Paramedics lead in crises, with police support for safety. | Collaborative model; authorised paramedics can direct police. | Paramedics assist clinicians. | Police-led, paramedics assist. | Paramedics take the lead. | Paramedics and police have similar emergency apprehension powers. | Police only in high-risk cases. | Clearly define paramedic and ambulance service roles in NZ. |
| **5. Specific Powers Granted to Paramedics** | Limited powers. No specific powers of detention. | Paramedics assist clinicians. | Limited powers. | Paramedics have detention powers. | Can be designated “authorised mental health practitioners” with examination powers. | Limited, under medical direction. | Paramedics assist. | Paramedics can detain and use reasonable force. | Paramedics have power of emergency apprehension for transport. | Limited, under medical oversight. | Expand paramedic powers in NZ. |
| **6. Legal Protections for Paramedics** | Limited. Relies on general criminal law provisions. | Paramedics protected in teams. | Protected when assisting police. | Strong legal protections when acting in good faith. | Protection for authorised persons acting in good faith. | Limited. | Police protections stronger. | Strong protections for authorised actions. | Protection for officials acting honestly and without recklessness. | Legal protections in limited scope. | Strengthen legal protections for paramedics in NZ. |
| **7. Role of Police** | Limited to high-risk situations. | Police assist in crisis teams. | Critical for detentions. | Police only for dangerous cases. | Can act on recommendation of authorised paramedic/practitioner. | Similar to NZ. | Significant role in detentions. | Police only for dangerous cases. | Similar emergency powers to paramedics; otherwise limited. | Police present in complex cases. | Clear guidelines for police in NZ needed. |
| **8. Cultural and Community Considerations** | Strong Māori focus. | Emphasis on diversity and community integration. | Less emphasis on cultural integration. | Focus on Aboriginal and Torres Strait Islander rights. | Principles include respect for culture and language. | Cultural sensitivity required. | Emphasis on cultural safety. | Community care integrated. | General principles of cultural sensitivity, but less specific than WA or NZ. | Strong cultural competency. | Increase paramedic training on intersection between cultural and mental health in NZ. |
| **9. Reducing Coercion** | Focus on voluntary care. | Least restrictive, voluntary options. | Focus on minimising coercion. | Focus on non-coercive treatment. | Strong emphasis on least restrictive care. | Emphasis on voluntary care. | Minimising coercion. | Non-coercive focus. | Strong rights-based principles in the Act. | Focus on reducing coercion. | Clear protocols needed for paramedics for non-coercive treatment in NZ. |
| **10. Oversight Mechanisms for Paramedics** | Limited. | Strong health service oversight. | Police or clinician oversight. | Paramedics report to health authorities. | Oversight via Chief Psychiatrist and Authorised Mental Health Services. | Limited. | Clinician oversight, limited. | Paramedics report to health. | Oversight is primarily through the ACAT legal framework. | Health service oversight strong. | Implement oversight in NZ. |
| **11. Private Ambulance Services** | Challenges integrating private services- dual reporting. | Public services only. | Public services only. | Fully publicly-funded, integrated private service. | Public service model. | Public services only. | Public services only. | Public services only. | Public service model. | Public services only. | Address public-private integration challenges in NZ by ensuring ambulance have operational integration and funded appropriately. |
| **12. Prescribing Authority** | Not granted to paramedics. | Not granted. | Not granted. | Not granted. | Not granted. | Not granted. | Not granted. | Not granted. | Not granted. | Granted to paramedics outside the Mental Health Act. | Follow UK model to ensure paramedic authorised prescribing enabled through the medicines regulation (United Kingdom Parliament, 2022). |

## Table 2: Summary of Recommendations

| **Framework Element** | **Recommendation** |
| --- | --- |
| **1. Criteria for Involuntary Detention** | Expand paramedic authority in New Zealand to assess risk and make detention decisions during crises. |
| **2. Emergency Detention & Transportation** | Grant paramedics in New Zealand legal authority to perform emergency detentions and transport in crises, similar to the Northern Territory model (Northern Territory Parliament, 1998). |
| **3. Integration of Services** | Strengthen the integration of paramedics with mental health services in New Zealand, incorporating paramedics into multidisciplinary crisis teams operated out of ambulance services, similar to Victoria (Victoria Parliament, 2022). Resource ambulance services to directly employ mental health clinicians to provide multi-disciplinary planning, responses and to support integration with mental health services. |
| **4. Role of Paramedics vs Police** | Clearly define the role of paramedics in mental health crisis interventions, with reduced reliance on police, and provide paramedics with sufficient legal tools and training to manage crises. |
| **5. Specific Powers Granted to Paramedics** | Expand the specific legal powers of paramedics in New Zealand to include independent decision-making in crisis management, aligned with models from Northern Territory (Northern Territory Parliament, 1998). |
| **6. Legal Protections for Paramedics** | Strengthen legal protections for paramedics in New Zealand, ensuring they are safeguarded when intervening in mental health crises, similar to the protections seen in the Northern Territory (Northern Territory Parliament, 1998). |
| **7. Role of Police** | Establish clear guidelines for the limited involvement of police in New Zealand mental health crises, restricting their role to situations involving significant risk or violence. |
| **8. Cultural and Community Considerations** | Enhance paramedic training in New Zealand, focusing on cultural competence and mental health care that aligns with Māori values and the importance of whānau involvement in crisis interventions. |
| **9. Reducing Coercion** | Develop clear protocols in New Zealand to ensure paramedics follow non-coercive practices in managing mental health crises, with a focus on voluntary care and reducing the use of coercion. |
| **10. Oversight Mechanisms for Paramedics** | Implement robust oversight mechanisms in New Zealand, ensuring paramedics are supported and accountable in mental health interventions, with health authority supervision similar to the frameworks in Victoria and Western Australia (Victoria Parliament, 2022) (Western Australia Parliament, 2014). |
| **11. Private Ambulance Services** | Address the challenges posed by New Zealand’s private ambulance services by ensuring operational integration with public health services, with appropriate funding and governance mechanisms in place to support paramedics’ expanded roles. |
| **12. Prescribing Authority** | Follow UK model to ensure paramedic authorised prescribing enabled through the medicines regulation (United Kingdom Parliament, 2022). |

## Appendix 1: Guiding Questions for Comparative Framework

The 12 domains of the comparative framework were guided by the following key questions:

1. **Criteria for Involuntary Detention and Treatment:** What are the legal thresholds for involuntary assessment or treatment, and who can initiate this process?
2. **Emergency Detention and Transportation:** Does the law grant specific powers to paramedics to detain and/ortransport a person in a mental health crisis?
3. **Integration of Mental Health and Crisis Services:** Does the legislation provide for formal integration or collaboration between ambulance services and mental health services (e.g., co-response teams)?
4. **Role of Paramedics versus Police in Crisis Interventions:** What is the legislated role of paramedics compared to police in responding to mental health emergencies?
5. **Specific Powers Granted to Paramedics:** Beyond transport, what specific clinical or legal actions are paramedics empowered to take?
6. **Legal Protections and Responsibilities of Paramedics:** Does the act provide specific legal protections or indemnity for paramedics acting in good faith?
7. **Role of Police in Mental Health Crises:** What is the defined role for police, and under what circumstances is their involvement mandated?
8. **Cultural and Community Considerations:** Does the legislation include specific provisions for culturally and linguistically diverse populations, particularly Indigenous peoples?
9. **Emphasis on Reducing Coercion:** Does the act explicitly prioritize patient rights, supported decision-making, and the use of the least restrictive interventions?
10. **Oversight Mechanisms for Paramedics:** What mechanisms are in place for the oversight, governance, and clinical review of paramedic actions in mental health crises?
11. **Challenges Posed by Private Ambulance Services:** How does the funding and governance model of the ambulance service (public vs. private) interact with the legislative framework?
12. **Prescribing Authority:** Does the legal framework allow for paramedics to prescribe or administer specific psychiatric medications?

## References

Northern Territory Parliament (1998) Mental Health and Related Services Act 1998.

United Kingdom Parliament (2022) Mental Health Bill 2022.

Victoria Parliament (2022) Mental Health and Wellbeing Act 2022.

Western Australia Parliament (2014) Mental Health Act 2014.
