# Supplementary material for: Paramedic powers in mental health crises: A comparative legal analysis
Source: Aust N Z J Psychiatry. 2025 Dec 7;60(2):184–90. doi: 10.1177/00048674251395412 (PMC12831802; doi:10.1177/00048674251395412)
Supplement: sj-pdf-1-anp-10.1177_00048674251395412 – Supplemental material for Paramedic powers in mental health crises: A comparative legal analysis [file sj-pdf-1-anp-10.1177_00048674251395412.pdf]

# Supplementary Document

Comparative Analysis of Mental Health Legislation

Table 1: Detailed Comparative Analysis

| **Framework Element** | **New Zealand (2024 Bill)** | **Victoria (AUS)** | **NSW (AUS)** | **WA (AUS)** | **QLD (AUS)** | **TAS (AUS)** | **SA (AUS)** | **NT (AUS)** | **ACT (AUS)** | **UK (2022 Act)** | **Key Considerations** |
|---|---|---|---|---|---|---|---|---|---|---|---|
| 1. Criteria for Involuntary Detention | Risk-based, rights-focused; least restrictive. | Least restrictive, voluntary first. | Similar to NZ. | Risk-based, with paramedic-detention powers. | Risk-based; requires authorised practitioner exam. | Medical authority only. | Similar to NSW. | Paramedics can detain on reasonable belief of risk. | Risk-based, immediate harm focus. | Risk-based. | Ensure clear paramedic decision powers in NZ. |
| 2. Emergency Detention & Transportation | Paramedics lack detention; only transport. | Transport under MH team direction. | Police assist detention. | Paramedics detain & transport to MH facility. | Authorised paramedics recommend; police apprehend. | Paramedics assist clinicians. | Police assist. | Paramedics detain independently. | Emergency apprehension powers. | Police assist; paramedics transport. | Expand NZ paramedic authority. |
| 3. Integration of Services | Conceptual integration only. | Strong PACER co-responses. | Police–clinician collaboration. | Multidisciplinary Crisis Teams. | "Authorised MH Services" model. | Needs closer collaboration. | Health-service integration. | Full paramedic integration. | Tribunal (ACAT) only. | Strong NHS integration. | Strengthen NZ ambulance–MH links. |
| 4. Role of Paramedics vs Police | Paramedics lead without special powers. | Co-response teams. | Police-heavy. | Paramedics lead; police for safety. | Authorised paramedics direct police. | Paramedics assist. | Police-led. | Paramedics lead. | Same emergency powers as police. | Police high-risk only. | Define NZ roles clearly. |
| 5. Specific Powers Granted to Paramedics | No detention powers. | Assist clinicians. | Limited. | Full detention powers. | Exam powers as MH practitioners. | Under medical direction. | Assist clinicians. | Detain + reasonable force. | Emergency apprehension only. | Under medical oversight. | Expand NZ powers. |
| 6. Legal Protections for Paramedics | Relies on general criminal law. | Team-based protection. | Protected when assisting police. | Strong good-faith immunities. | Good-faith protection for authorised. | Limited. | Strong police protections. | Good-faith immunities. | Honest/reckless standard. | Limited. | Bolster NZ protections. |
| 7. Role of Police | High-risk only. | Co-response support. | Critical detention role. | Only dangerous cases. | Act on paramedic recommendation. | Similar to NZ. | Significant detention role. | Dangerous cases only. | Mirrored paramedic powers. | Complex cases. | Clear NZ guidelines. |
| 8. Cultural & Community Considerations | Strong Māori focus. | Community integration emphasis. | Less cultural focus. | Aboriginal/Torres Strait Islander rights. | Respect culture/ language. | Cultural sensitivity needed. | Cultural safety emphasis. | Community care integration. | General cultural principles. | Strong competency. | Increase Māori-focused training. |
| 9. Reducing Coercion | Voluntary-first. | Least restrictive. | Minimise coercion. | Non-coercive treatment. | Least restrictive emphasis. | Voluntary focus. | Minimise coercion. | Non-coercive focus. | Rights-based Act principles. | Reduce coercion. | Protocols for non-coercion. |
| 10. Oversight Mechanisms for Paramedics | Very limited. | Health-service oversight. | Police/clinician oversight. | Report to health authority. | Chief Psychiatrist oversight. | Limited. | Clinician oversight. | Report to health. | ACAT framework. | Strong oversight. | Implement NZ oversight. |
| 11. Private Ambulance Services | Dual-reporting challenges. | Public-only. | Public-only. | Publicly funded integrated. | Public model. | Public-only. | Public-only. | Public-only. | Public model. | Public-only. | Resolve NZ public–private issues. |
| 12. Prescribing Authority | None. | None. | None. | None. | None. | None. | None. | None. | None. | Paramedic prescribing outside Act. | Follow UK medicines regulation. |

Table 2: Summary of Recommendations

| Framework Element | Recommendation |
|---|---|
| 1. Criteria for Involuntary Detention | Expand NZ paramedic authority to assess risk and detain during crises. |
| 2. Emergency Detention & Transportation | Grant NZ paramedics emergency detention & transport powers, as in NT (Northern Territory Parliament 1998). |
| 3. Integration of Services | Embed paramedics in MH co-response teams (PACER model) and fund ambulance-employed MH clinicians (Victoria Parliament 2022). |
| 4. Role of Paramedics vs Police | Legally define paramedic lead role, reduce police reliance, and mandate paramedic MH training. |
| 5. Specific Powers Granted to Paramedics | Authorise NZ paramedics for independent crisis decisions, mirroring NT (Northern Territory Parliament 1998). |
| 6. Legal Protections for Paramedics | Codify good-faith immunities for NZ paramedics, as in NT (Northern Territory Parliament 1998). |
| 7. Role of Police | Restrict police to major risk scenarios in NZ, with clear activation criteria. |
| 8. Cultural and Community Considerations | Enhance Māori-centric training and whānau engagement for NZ paramedics. |
| 9. Reducing Coercion | Implement NZ protocols for voluntary-first, non-coercive MH crisis care. |
| 10. Oversight Mechanisms for Paramedics | Create robust NZ oversight under health authorities, akin to Vic (Victoria Parliament 2022) & WA (Western Australia Parliament 2014). |
| 11. Private Ambulance Services | Develop NZ governance/funding to integrate private ambulance into public MH crisis system. |
| 12. Prescribing Authority | Introduce paramedic prescribing via NZ medicines regulation, following UK (United Kingdom Parliament 2022). |

## The 12 domains of the comparative framework were guided by the following key questions:

1. **Criteria for Involuntary Detention and Treatment:** What are the legal thresholds for involuntary assessment or treatment, and who can initiate this process?
2. **Emergency Detention and Transportation:** Does the law grant specific powers to paramedics to detain and/or transport a person in a mental health crisis?
3. **Integration of Mental Health and Crisis Services:** Does the legislation provide for formal integration or collaboration between ambulance services and mental health services (e.g., co-response teams)?
4. **Role of Paramedics versus Police in Crisis Interventions:** What is the legislated role of paramedics compared to police in responding to mental health emergencies?
5. **Specific Powers Granted to Paramedics:** Beyond transport, what specific clinical or legal actions are paramedics empowered to take?
6. **Legal Protections and Responsibilities of Paramedics:** Does the act provide specific legal protections or indemnity for paramedics acting in good faith?
7. **Role of Police in Mental Health Crises:** What is the defined role for police, and under what circumstances is their involvement mandated?
8. **Cultural and Community Considerations:** Does the legislation include specific provisions for culturally and linguistically diverse populations, particularly Indigenous peoples?
9. **Emphasis on Reducing Coercion:** Does the act explicitly prioritize patient rights, supported decision-making, and the use of the least restrictive interventions?
10. **Oversight Mechanisms for Paramedics:** What mechanisms are in place for the oversight, governance, and clinical review of paramedic actions in mental health crises?
11. **Challenges Posed by Private Ambulance Services:** How does the funding and governance model of the ambulance service (public vs. private) interact with the legislative framework?
12. **Prescribing Authority:** Does the legal framework allow for paramedics to prescribe or administer specific psychiatric medications?

# References


Northern Territory Parliament (1998). *Mental Health and Related Services Act 1998*. Northern Territory, Australia. URL: https://legislation.nt.gov.au/en/Legislation/MENTAL-HEALTH-AND-RELATED-SERVICES-ACT-1998.

United Kingdom Parliament (2022). *Mental Health Act 2022*. United Kingdom. URL: https://www.mind.org.uk/about-us/our-policy-work/mental-health-act-reform/.

Victoria Parliament (2022). *Mental Health and Wellbeing Act 2022*. Victoria, Australia. URL: https://www.legislation.vic.gov.au/in-force/acts/mental-health-and-wellbeing-act-2022/001.

Western Australia Parliament (2014). *Mental Health Act 2014*. Western Australia, Australia. URL: https://www.parliament.wa.gov.au/parliament/commit.nsf/(Report+Lookup+by+Com+ID)/AD292116C942943E48257D9D0009C9E6/$file/Discussion+Paper+Final+PDF.pdf.
